# The New Cottage Hospital at Exmouth

**Published:** 1903-06-20

**Authors:** 


### THE NEW COTTAGE HOSPITAL AT EXMOUTH.

THERE is now an excellent prospect of the new Cottage Hospital at Exmouth being opened free from debt this summer. The new building will cost over £3,000, and incidental expenses will swallow up close on another £1,000. The committee had about £2,500 in hand prior to last week's bazaar, and it is hoped to obtain £600 for the existing institution. The bazaar was on an elaborate scale, and the opening ceremony was performed by Lady Kennaway, and Sir John Phear, who presided, made an urgent appeal for help to carry out the work so generously commenced by the late Mrs. Hume Long. The stalls were representative of Windsor Castle, the Houses of Parliament, the verandah of the new hospital, the Tower of London, etc., and the committee and those who worked so arduously must have been gratified at the success which rewarded their efforts. Something like 3,000 persons attended during the three days, and no less than £1,085 was taken, of which it is hoped £1,000 will remain on behalf of the funds.

